# Refractory hypotension due to intraoperative hypothermia during spinal instrumentation

**DOI:** 10.4103/0019-5049.60500

**Published:** 2010

**Authors:** Ponniah Vanamoorthy, Mihir P Pandia, Parmod K Bithal, Sebastian S Valiaveedan

**Affiliations:** Department of Neuroanesthesiology, All India Institute of Medical Sciences, New Delhi, India

**Keywords:** Hypothermia, hypotension, spinal surgery

## Abstract

We report a case of inadvertent hypothermia leading to severe hypotension resistant to high dose vasopressors, which responded to temperature correction in a patient undergoing spinal instrumentation surgery. A 60-year-old female developed severe hypotension during spinal instrumentation surgery. After review of all factors it was found to be secondary to hypothermia. The patient did not respond to high dose vasopressors. However, when normothermia was restored she recovered uneventfully. Patients undergoing lengthy spinal procedures in prone position are vulnerable to develop hypothermia and consequent cardiovascular depression so adequate measures should be taken to prevent hypothermia.

## INTRODUCTION

Inadvertent hypothermia is by far the most common perioperative thermal disturbance, resulting from a combination of impaired thermoregulation and exposure to a cold atmosphere.[1] Severe cardiovascular depression and pulseless electrical activity has been reported in victims of accidental hypothermia but such incidents are rare in the operating room because of temperature monitoring and preventive measures.[2] Prolonged spinal surgery under general anaesthesia can have number of complications.[3] We report a case of severe hypotension, refractory to standard treatment secondary to inadvertent hypothermia during spinal instrumentation for Grade II L5 S1 spondylolisthesis.

## CASE REPORT

A 60-year-old female weighing 62 kg with Grade II L_5-_S_1_ spondylolisthesis was scheduled for L_5_ laminectomy, reduction and instrumentation. She was a known hypertensive for nine years and her blood pressure (BP) was controlled on amlodipine 5 mg and atenolol 50 mg orally, once daily. On the preoperative visit, her BP and HR were found to be 130/80 mm Hg and 78 beats per minute respectively. All other investigations were normal. She had no clinical evidence of any end organ dysfunction or limitation of physical activity.

The patient received diazepam 5 mg per orally, the night before surgery, atenolol 50 mg, amlodipine 5 mg, per orally, two hours before surgery and glycopyrrolate 0.2 mg intramuscularly half hour before surgery. Hydrocortisone 200 mg intravenously was given before induction as she was on long term steroid therapy.

General anaesthesia was induced with fentanyl 150 *μ*g and thiopentone 250 mg. Rocuronium 50 mg was administered to facilitate endotracheal intubation. Anaesthesia was maintained with 66% nitrous oxide in oxygen, isoflurane, fentanyl and vecuronium. Continuous monitoring of ECG, pulse oximetry, NIBP, IBP (left radial artery) and nasopharyngeal temperature was done. The patient was positioned prone on the Wilson's frame. After the patient was made prone, there was malfunctioning of temperature probe and temperature monitoring was not possible. The patient was covered with surgical drapes and received warm intravenous fluids. The patient remained haemodynamically stable until the instrumentation was completed which lasted for six hours. The estimated blood loss was 1500 ml. She received 4000 ml crystalloid, 1000 ml colloid and four units of packed red blood cells after which the haematocrit was 33% and there was a total urine output of 1000 ml. During closure of the surgical wound she gradually developed hypotension with a systolic BP 80 mm Hg.

A fluid challenge of 500 ml Ringer Lactate was given without much benefit and the SBP decreased to 70 mm Hg. Mephenteramine 6 mg intravenous bolus was given and dopamine 10 mcg/kg/min infusions started. Noradrenaline infusion was started when the BP continued to be low despite the dopamine infusion. Hydrocortisone 200 mg was administered for a possible adrenal insufficiency. In spite of dopamine (up to 10 mcg/kg/min) and noradrenaline (up to 20 mcg/min) infusions, SBP remained below 60 mm Hg over the next 30 minutes. Adrenaline infusion 5 mcg/min were also added without much benefit. First bolus of adrenaline 0.1 mg was given when SBP was 40 mm Hg and subsequently repeated every time when the SBP was<60 mm Hg. The arterial blood gas (ABG) analysis at this time revealed a metabolic acidosis with pH of 7.205.

The closure was completed quickly. There was no improvement in hypotension even after turning the patient supine. Right internal jugular vein was cannulated and the CVP was found to be 18 mm Hg with good wave form. A thorough systemic examination revealed hypothermia with a nasopharyngeal temperature of 31°C. The patient was actively rewarmed with forced air warmer and warm IV fluids (40-42°C). ABG analysis revealed worsening of metabolic acidosis with a pH of 7.175. Soda bicarbonate 160 meq was given. Twelve lead electrocardiogram (ECG) revealed no abnormalities and cardiac enzymes were normal (troponin I). Bolus doses of adrenaline 0.1 mg were used whenever the SBP fell below 60 mm Hg and the BP was maintained between 70 and 80 mmHg.

In the intensive care unit (ICU) she received ventilator assistance. Despite infusions of dopamine and nor adrenaline patient needed boluses of adrenaline to maintain blood pressure. Patient required 28 boluses of 0.1 mg adrenaline each during the periods of hypotension. In spite of adrenaline boluses SBP was never above 85 mm Hg. Residual neuromuscular blockade was reversed after about two hours when the core body temperature measured at nasopharynx increased to 33.6°C. Over next 1 hour she started responding to commands and the SBP was maintained between 100 and 110 mm Hg. Rewarming was continued until 36°C, which further raised BP to 114/70 mm Hg. Noradrenaline and dopamine infusions were tapered and stopped within 30 minutes after the temperature was above 35°C. Further postoperative course was uneventful.

## DISCUSSION

Anaesthesia for major spinal surgeries in the prone position presents a number of challenges. Severe unexpected hypotension following prone position has been reported.[4–7] Decrease in the cardiac output, stroke volume, cardiac index occur in varying degrees when a patient is positioned prone depending on the cardiovascular status of the patient and positioning systems (Wilson Frame, Siemens, Bolsters etc).[8]

Intraoperative hypothermia can occur during major spinal surgeries. Long duration of surgery, massive blood loss requiring transfusion, no active rewarming measure, elderly patients and patients with low body mass are some of the factors which predispose to hypothermia.[9] Our patient developed hypothermia because of prolonged surgery, and large amount of intravenous fluid infusion, blood transfusion and insufficient temperature control because of absence of temperature monitoring.

The most common causes of hypotension during spinal surgeries are increased anaesthetic depth, hypovolemia secondary to blood loss and impaired venous return. We excluded other rare causes of hypotension such as myocardial infarction, pulmonary embolism, metabolic and electrolyte abnormalities, adrenocortical insufficiency, drug toxicity, anaphylaxis, tension pneumothorax and mismatched blood transfusion. The abnormal observation in our patient was hypothermia (31°c). We believe that the hypotension that was refractory to vasopressors therapy was secondary to hypothermia. Severe cardiovascular depression leading to pulse less electrical activity has been reported following severe hypothermia.[10] The body's initial response to cold stress is to generate and conserve heat via activation of the sympathetic nervous system.

Blood pressure rises as a result of peripheral vasoconstriction and increased cardiac output. In contrast with progression to moderate to severe hypothermia, the initially elevated catecholamines return to baseline as the patient enters a state of globally depressed organ function and hypothermia exhibit progressive decreases in both mean arterial pressure (MAP) and cardiac output.[11,12] Although our patient did not respond to vasopressors, when hypothermic, after rewarming the blood pressure could be restored and we could taper and stop the vasopressors within 30 minutes. With improvement in temperature, the BP improved. The patient was on calcium channel blockers and beta blockers, both synergistic myocardial depressants. The synergistic cardiovascular depressant effect of amlodipine and atenolol might have augmented the cardiovascular depression caused by hypothermia. There is also evidence that beta blockers have central effects which can augment induced hypothermia.[13]

Under anaesthesia, thermoregulation is impaired and even with routine perioperative thermal care, approximately one half of patients develop hypothermia. When hypothermia causes cardiovascular depression vasopressors will not be effective in restoration of blood pressure and active external warming forms the main stay of treatment.[14] External warming may not be effective in patients undergoing spinal surgery in the prone position. If catastrophe occurs in this setting it is even more difficult to resuscitate the patient as vital time will be lost in making the patient supine.

Our case emphasises the need for continuous temperature monitoring and adequate measures to prevent hypothermia in patients undergoing lengthy spinal procedures in prone position. To conclude, we present a case of refractory hypotension secondary to hypothermia in a patient undergoing spinal instrumentation surgery in the prone position.
